# Oligometastatic recurrence of an oesophageal adenocarcinoma at a chest drain site following radical treatment: palliative treatment or resection?

**DOI:** 10.1007/s12328-018-0890-0

**Published:** 2018-08-25

**Authors:** David Wen, Elena Collantes, Bruno Sgromo

**Affiliations:** 1 University of Oxford Medical School, John Radcliffe Hospital, Oxford, UK; 2 Department of Cellular Pathology, John Radcliffe Hospital, Oxford University Hospitals NHS Foundation Trust, Oxford, UK; 3 Department of Upper GI Surgery, Churchill Hospital, Oxford University Hospitals NHS Foundation Trust, Oxford, UK

**Keywords:** Oligometastasis, Oesophageal cancer, Oesophagectomy

## Abstract

A 62-year-old female patient diagnosed with oesophageal adenocarcinoma underwent radical treatment consisting of neoadjuvant chemotherapy and oesophagectomy with no major complications. Eleven months later, she re-presented with a mass at one of the chest drain sites. A PET-CT scan and biopsy demonstrated this to be a single recurrence of the oesophageal adenocarcinoma. Excision of the metastatic lesion was considered as per metachronous single site metastasis. However, the operation was postponed due to acute kidney injury. Restaging after 6 weeks revealed progressive metastatic disease. The patient underwent palliative therapy and passed away soon after. Oesophageal cancer recurrence has a very poor prognosis, and factors such as the disease-free interval, site of recurrence and tumour pathological factors must be considered when stratifying for suitability for metastasectomy. A period of watchful waiting followed by restaging is essential to rule out patients with indolent metastatic disease.

## Introduction

First proposed by Hellman and Weichselbaum in 1995 [1], oligometastases are metastases that are limited in number, site and metastatic potential, and can thus potentially be cured with radical treatment [2]. Management of oligometastatic recurrence after oesophageal cancer is currently under debate, with long-term survival after metastasectomy being variable.

There are no documented cases of oesophageal adenocarcinoma seeding to a chest drain site. However, metastasectomy ± adjuvant therapy of single PEG site recurrences from direct seeding of head and neck cancers has been shown to achieve long-term survival in patients [3–6]. Additionally, port site recurrences of upper GI carcinomas have also been treated successfully with metastasectomy followed by adjuvant chemotherapy [7].

Although seldom attempted, reports have shown that long-term survival following oesophageal cancer recurrence can be achieved after resection of solitary oligometastases in the lung [8], and adrenal gland [9]. A number of case series have demonstrated that selected patients with lung oligometastases treated with metastasectomy (± adjuvant or neoadjuvant treatment) have a greater long-term survival on average than those who were not selected for resection and palliated [8, 10]. There have also been documented cases where resection has been attempted in the liver, although the outcomes following these attempts have been poor, with only a small chance of success [10, 11].

The outcomes following resection of liver metastases from gastric cancer have also been investigated [12]. This study showed that patients who underwent resection of metachronous oligometastases achieved longer survival compared to patients who underwent resection of gastric cancer with synchronous metastasectomy [12].

To the best of our knowledge, this is the first report of a metachronous recurrence of an oesophageal adenocarcinoma at a chest drain site. Due to the initial presentation as a single chest wall oligometastasis shown by CT and PET/CT, it was thought that seeding of the cancer to the chest drain site was solitary and had occurred directly, in a fashion similar to port site metastasis, where direct seeding is thought to be a more likely mode of transmission than haematogenous spread [13, 14]. Additionally, the patient had been in remission for almost a year (11 months since surgery). For these reasons, and based on the encouraging results mentioned above, a wide local excision with curative intent was thought to be suitable for our patient.

## Case report

A 62-year-old obese lady presented with a 2-month history of dysphagia for solid foods which had worsened over the past 2 weeks, progressing to odynophagia. She was otherwise well and not on any medications. She did not smoke, and only drank moderately.

**Fig. 1:**
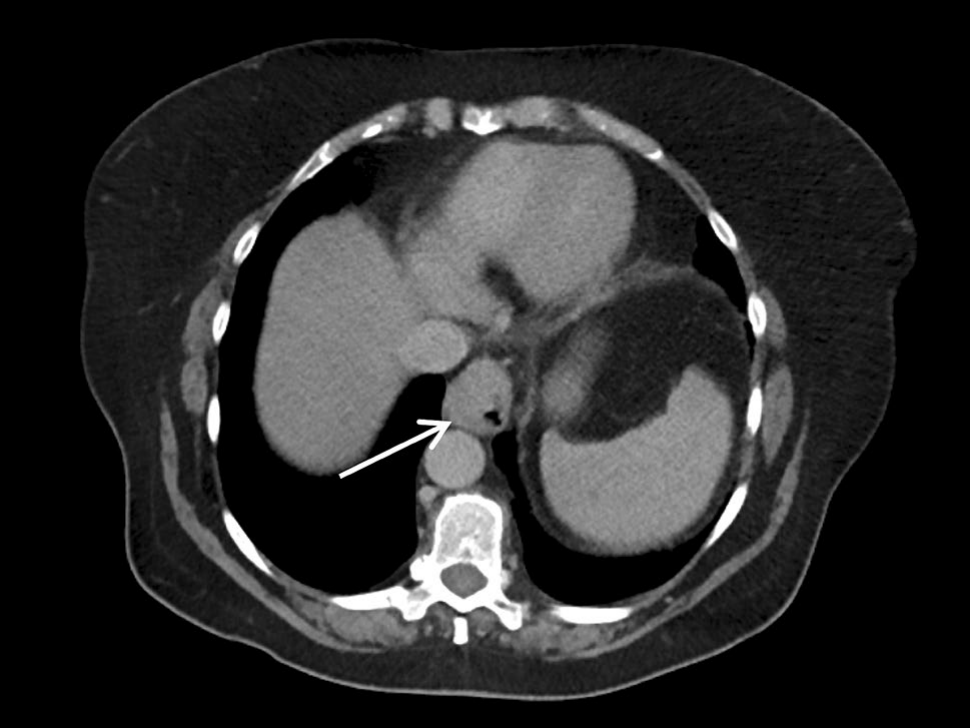
CT scan showing oesophageal cancer

Oesophago-gastroduodenoscopy (OGD), biopsy and CT revealed a 5-cm-long circumferential, invasive and poorly differentiated adenocarcinoma at the GOJ (Fig. 1). Subsequent PET-CT showed no evidence of FDG avid local or distant spread giving a clinical (cTNM) stage of IIA (cT3 N0 Mx) with a mildly avid standardised uptake value (SUV) of 5. Staging laparoscopy confirmed no peritoneal disease, so a feeding jejunostomy was placed. An endoscopic ultrasound (EUS) was not carried out due to the stricturing cancer and as it was thought to have been unlikely to lead to any changes in the treatment plan.

At the time of her initial presentation, our trust protocol for patients with a tumour staged as being T2 N0 or above was neoadjuvant chemotherapy without radiotherapy. She underwent three cycles of epirubicin, cisplatin and capecitabine (ECX) neoadjuvant chemotherapy. Restaging with PET-CT 3 months later showed that the cancer stage remained the same at IIA and that there was a marginal reduction of the SUV to 4.4.

A hybrid Ivor Lewis oesophagectomy with laparoscopic gastric mobilisation was performed with no intra-operative complications. Lymphadenectomy was performed en bloc of stations 4R, 7, 8L, 8M, 9, 10L, 10R, 15–20 (AJCC 7th Esophageal Cancer Staging Manual, 7th Edition). As the specimen was removed through the thoracotomy site, a wound protector was not used. No spillage or perforation of the tumour was noted at time of surgery. The oesophagogastric anastomosis was performed at the level of the thorax, above the azygous vein and 3 cm below the thoracic inlet. Two chest drains were placed as per standard practice. She recovered with no major post-operative complications and was discharged home day 8 post-operative.

**Fig. 2:**
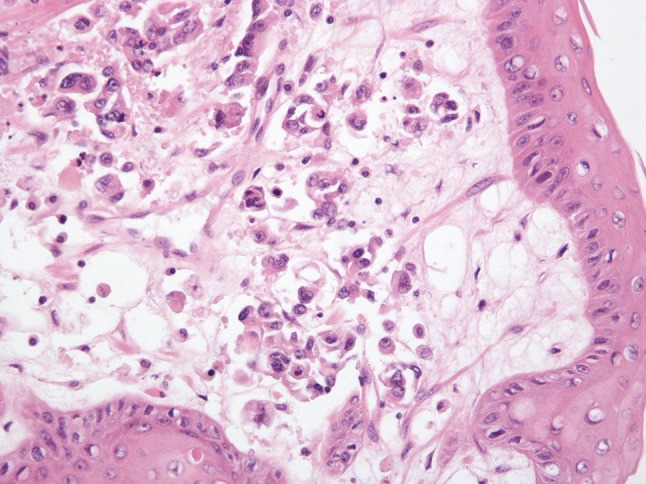
H&E stain of oesophageal primary tumour

The pathological examination demonstrated a ypT4a N2 (3/25) L1 V1 M0 R1 tumour, which was not present in the proximal oesophageal margin block and was therefore at least 5 mm from the proximal surgical resection margin. However, the pathological circumferential resection margin (CRM) was tumour positive (R1), defined as tumour involvement within 1 mm of the surgical resection margin [British Royal College of Pathology (http://www.rcpath.org)]. The tumour was also seen to be spreading along the submucosa and subserosa of the stomach. Three of 25 lymph nodes examined were tumour positive. There were no regressive changes from neoadjuvant chemotherapy corresponding to a Mandard tumour regression grade of 5. Histologically, the tumour was a poorly differentiated adenocarcinoma (diffuse type) with scattered signet ring cells and overlying reactive squamous mucosa (Fig. 2). Immunohistochemistry showed positive staining with CK7.

Due to the R1 staging, she subsequently underwent adjuvant chemoradiotherapy (45 Gy in 25 fractions with four cycles of carbotaxol), which was standard local practice for a R1 specimen.

Eleven months after the oesophagectomy, our patient re-presented with a non-tender and irreducible mass in the right chest wall at the site of the previous chest drain. There were no further lesions or ascites evident on clinical examination.

**Fig. 3:**
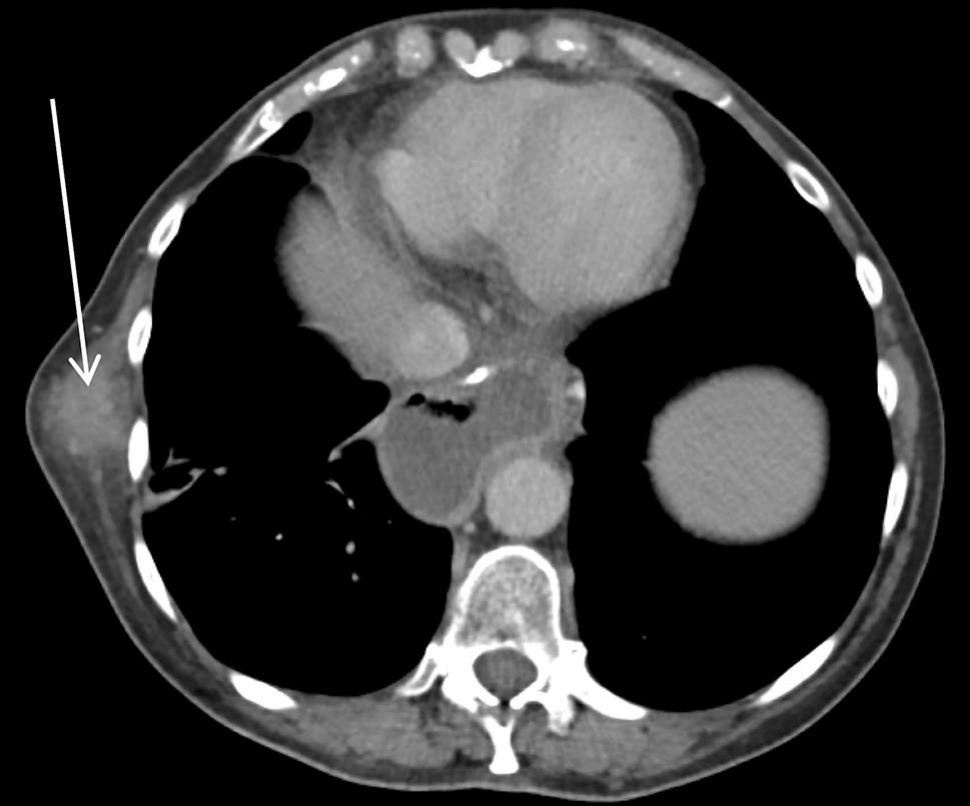
CT scan showing right lateral chest wall mass

**Fig. 4:**
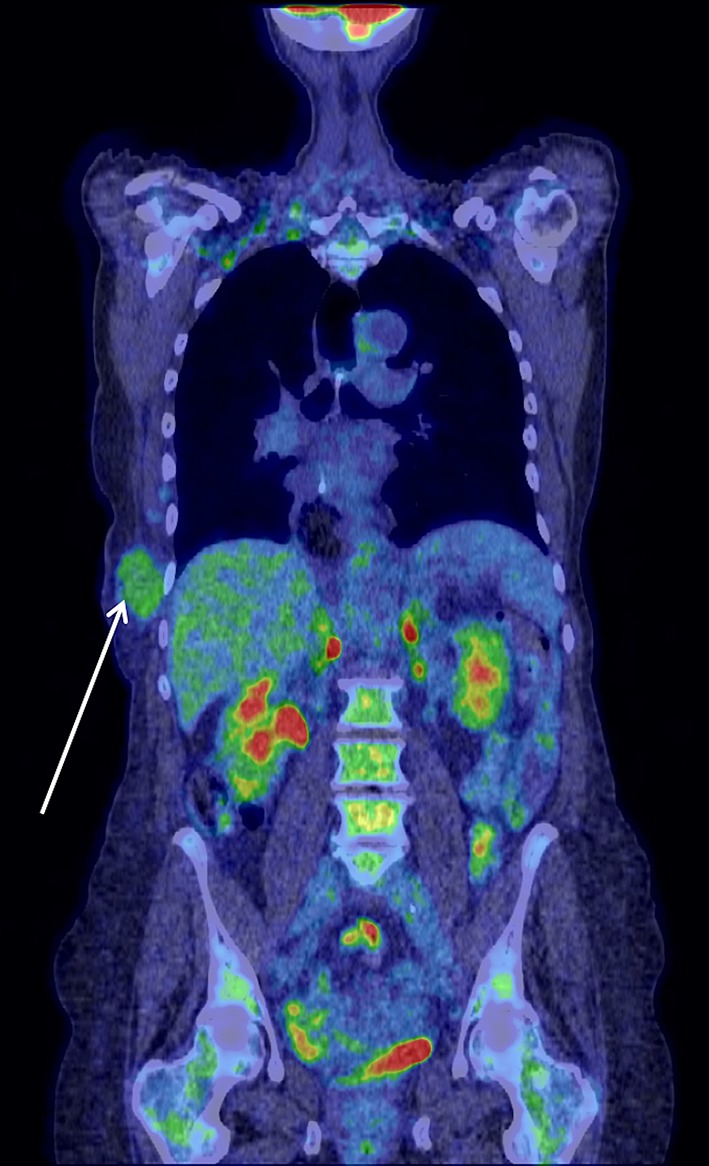
A PET scan showing a mildly FDG avid recurrence in the right lateral chest wall, with no FDG avid metastases or ascites visible

**Fig. 5:**
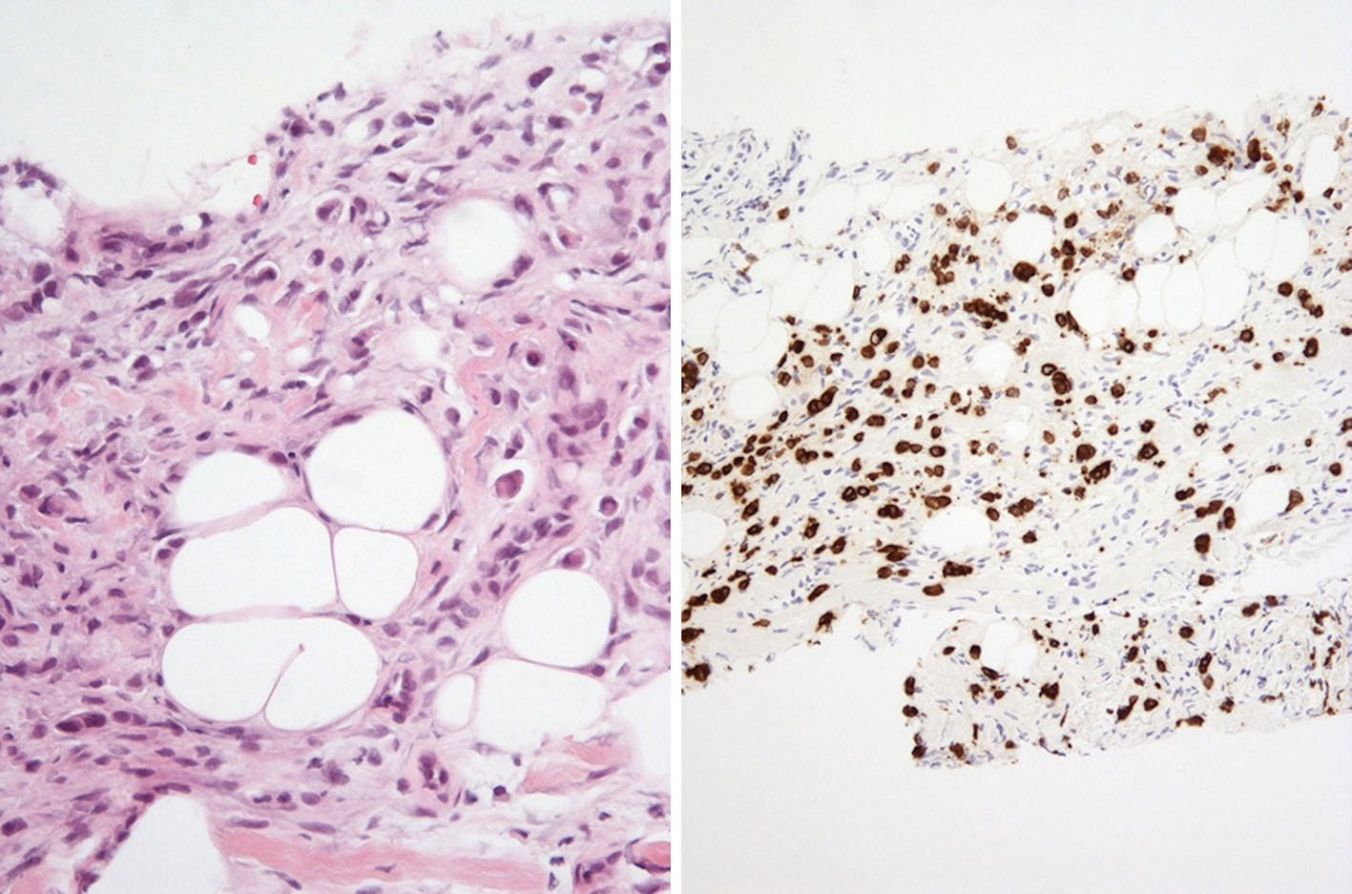
H&E stains of the chest wall lesion (left) with positive staining for CK7 (right)

CT scans confirmed the presence of a chest wall mass (Fig. 3). A PET scan showed a mildly FDG avid recurrence in the right lateral chest wall, with no FDG avid metastases or ascites elsewhere, suggesting that the mass was a single site recurrence (Fig. 4). A core biopsy of the mass showed fibro-fatty tissue infiltrated by a diffuse-type adenocarcinoma. Immunohistochemistry showed positive staining for CK7, and showed no evidence of TTF1, CD45, oestrogen or progesterone receptors (Fig. 5). The morphology and immunophenotype were consistent with the previous oesophageal primary tumour with both samples showing proliferation of epithelioid cells with moderate eccentric eosinophilic cytoplasm, and some signet ring cells with occasional gland formation in the primary location. Additionally, FDG avidity was similar to the initial oesophageal tumour. Due to these features, the mass was likely to be a recurrence of the original oesophageal cancer. Along with the histological and radiological findings, the patient had no respiratory symptoms, including cough or haemoptysis, making lung cancer unlikely.

**Fig. 6:**
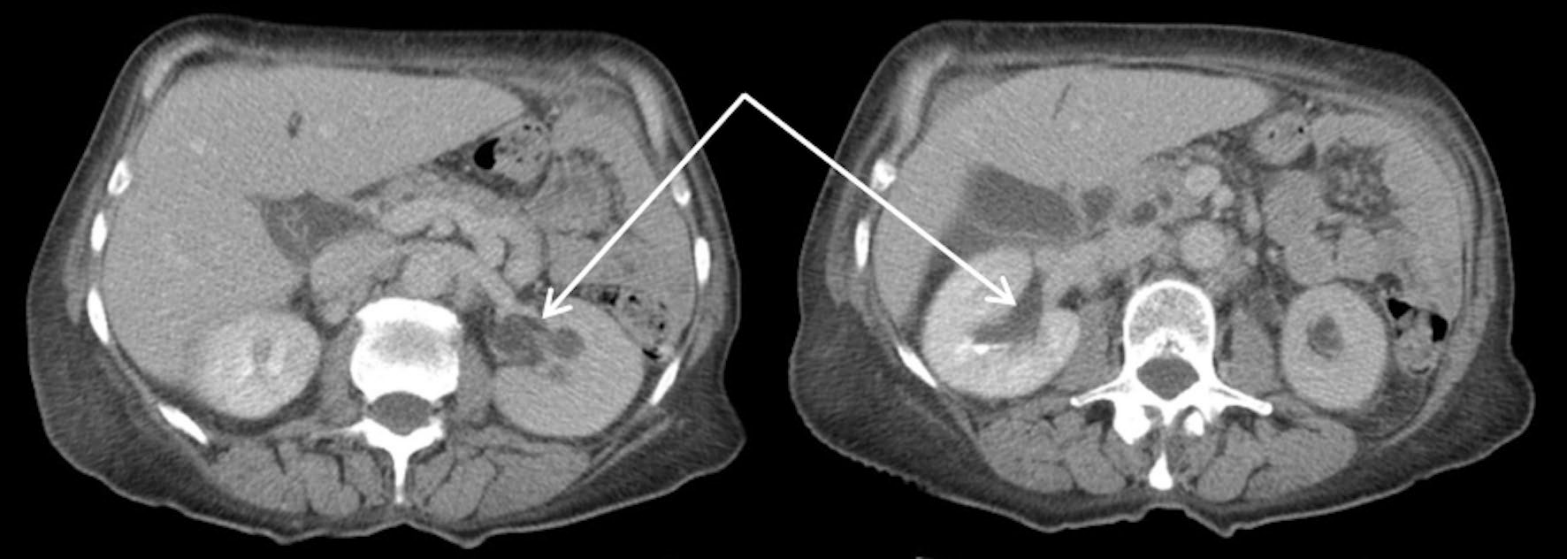
CT scan showing hydronephrosis of the left and right kidneys

Following MDT discussion, it was decided to proceed with surgical resection after considering the disease-free interval (DFI) of 11 months, and that the recurrence appeared to be a solitary oligometastasis which has seeded directly. This was favoured over radical chemoradiotherapy as given the age and life expectancy of our patient, we believed that surgical resection would give her the best chance of long term survival. The resection of the metastasis did not take place as our patient was found to have acute kidney injury (AKI) during the pre-operative assessment. Ultrasound and CT scans demonstrated moderate functional bilateral hydronephrosis (Fig. 6) with no evidence of urinary lithiasis or periureteral masses. Double J stents were inserted bilaterally with no complications but did not relieve the hydronephrosis. The planned excision of the chest wall lesion was postponed until bilateral nephrostomies were inserted under radiological guidance and renal function returned to normal.

**Fig. 7:**
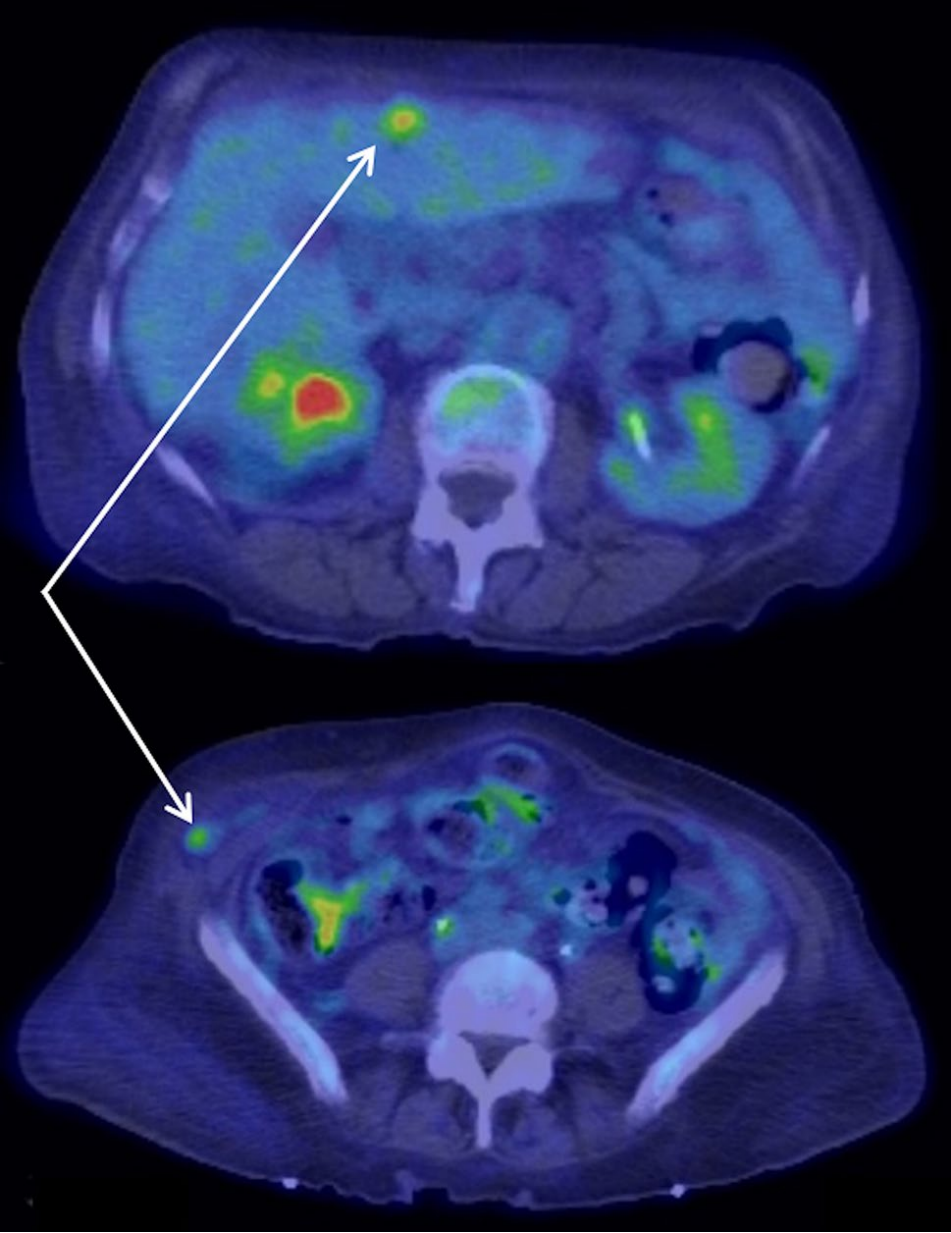
A PET scan showing a focus of increased FDG uptake in the liver (top) and in a Spigelian hernia (bottom)

Six weeks after presenting with a chest wall mass, and after the resolution of the AKI, our patient was restaged with a PET-CT scan. This revealed a focus of increased FDG uptake in the liver and also in an incidental Spigelian hernia (Fig. 7). It also showed increasing peritoneal free fluid and an increase in size from 3 to 5 cm of the chest wall lesion. Considering the rapid progression of the recurrent disease, she was managed non-operatively with palliative therapy. She passed away 2 months later.

## Discussion

Management of oligometastatic recurrence after oesophageal cancer is controversial, with long-term survival after metastasectomy being variable. When selecting suitable candidates to undergo resection, one may wish to consider prognostic factors such as the disease-free interval (DFI), site of recurrence, and the pathological characteristics of the primary tumour.

In a case series by Chen et al. [8], five patients with metachronous oesophageal cancer metastases to the lung underwent pulmonary metastasectomy. One patient had widespread recurrence afterwards in the neck, lung and brain, and subsequently died 11 months post op. This patient had the shortest DFI from oesophagectomy to initial re-presentation (13 months). The four other patients had longer DFIs (21–59 months) and achieved long-term survival after metastasectomy. Similar findings have been noted in another case series which examined hepatectomy in patients with metachronous liver oligometastases from oesophageal carcinoma [11], suggesting that the DFI may be a potential marker for long-term outcome following metastasectomy. A shorter DFI also correlated with a smaller number of metastases at re-presentation, and the authors suggest these patients as being good candidates for metastectomy; all patients with single metastases in these reports achieved long-term survival after this treatment [8, 11].

It has been reported that resection of metachronous oligometastases in the lung increases the average survival of selected and suitable patients [10]. However, resection of metachronous oligometastases recurring in non-hepatic, non-pulmonary sites did not alter the natural disease course and conferred no survival benefit. Metastasectomy for recurrence in the lung conferred the most favourable prognosis when compared with metastasectomy for recurrence in the liver, and recurrence in non-pulmonary, non-hepatic sites. Thus, location of initial recurrence may also have prognostic implications [9, 15], and along with the DFI, may reflect tumour biology.

At re-presentation, our patient had a solitary mass in the chest wall with no other lesions or ascites detectable, and so it initially appeared to be an oligometastasis. We considered our patient’s metachronous single site metastasis to be similar to a PEG or port site metastasis from direct transmission, hence she was considered for surgery. However, the pathological features of her tumour were unfavourable for long-term survival [12, 16], particularly if her metastases had been blood borne or were able to spread in any other way, which it eventually did, 6 weeks after re-presentation. It is possible that the tumour invaded into the stomach wall like a scirrhous carcinoma, eventually resulting in small volume peritoneal dissemination and may have been a cause of the AKI and hydronephrosis. Poor prognostic features for survival included, at pathologic staging: T4a, poor differentiation, involved nodes (3/25), a positive CRM after resection (R1), and a poor response to neoadjuvant therapy (Mandard 5). It is noted that our patient’s cancer was under-staged by CT and PET-CT (cT3 N0 Mx vs ypT4a N2 M0 R1). An EUS scan was not feasible due to the presence of a malignant stricture. EUS would have improved the accuracy of staging, but it would have not changed the curative treatment plan according to our MDT protocol at the time.

The delay needed to optimise her renal function led to an important change in the treatment plan, ultimately avoiding unnecessary surgical intervention. As illustrated by this case, a period of active monitoring and restaging is essential when dealing with oligometastatic recurrence of oesophageal cancer. There are no reports or studies recommending a specific waiting time after initial re-presentation before aggressive treatment. For our patient, 6 weeks from initial re-presentation were enough for the widespread metastases to be detected. The outcomes for our patient would have been dismal if we proceeded immediately with a wide surgical excision, as she not only had an oligometastasis from direct seeding, but also initially unrecognised blood borne and transcoelomic metastases in the peritoneum. Further staging with laparoscopy and thoracoscopy may have a role in these situations too, as dissemination may initially be difficult to identify only with PET-CT. However, our patient’s AKI prevented us from doing this early on.

Evidence from previous reports have suggested good long-term survival for oesophageal cancer recurrence if the metastases are single and metachronous with a significant DFI, and also occurring in sites such as the lung. As shown by our case, an 11-month DFI was not long enough to predict a truly solitary recurrence and potential long-term survival after resection. A longer DFI may be required and more studies are required to delineate an estimate for a positive prognosis. By chance, our case also highlighted that an assessment period following recurrence should be adhered to, allowing metastases which are undetectable through imaging to be excluded. The precise length of this period is also yet to be determined.
